# Impact of diabetic foot ulcers on mortality risk: Lessons from a 35-year Brazilian cohort followed at an association of people living with diabetes

**DOI:** 10.20945/2359-4292-2026-0121

**Published:** 2026-10-11

**Authors:** Carlos Antonio Negrato, Hermelinda Cordeiro Pedrosa, Letícia Alcântara Chaparim, Monize Ferreira Catelli, Lucas Casagrande Passoni Lopes

**Affiliations:** 1 Faculdade de Medicina de Bauru, Universidade de São Paulo, Bauru, SP, Brasil; 2 Unidade de Endocrinologia, Hospital Regional de Taquaritinga, Brasília, DF, Brasil

**Keywords:** Diabetes mortality, diabetic foot ulcers, Brazil

## Abstract

**Objective:**

To evaluate the impact of diabetic foot ulcers (DFUs) on mortality risk in a Brazilian cohort of individuals followed at an association of people living with diabetes mellitus.

**Subjects and methods:**

This retrospective, population-based epidemiological study assessed the impact of DFUs on mortality risk in a cohort of Brazilian individuals with types 1 and 2 diabetes mellitus. Data were obtained from medical records covering the period from 1990 to 2024 and included sociodemographic, clinical, laboratory, and therapeutic variables. Survival analyses were conducted using Cox proportional hazards models, with death as the event of interest, and were adjusted for potential confounders.

**Results:**

Among the 382 participants included in the final analysis, DFUs, amputations, and diabetic peripheral neuropathy were associated with increased mortality risk by 89%, 123%, and 37%, respectively. Peripheral artery disease showed a modest, but not statistically significant, increase in mortality risk. Patients with DFUs who were not using oral antidiabetic drugs had a 165% higher mortality risk, and those with prior amputations and poor fasting glucose levels had a 41% increased mortality risk.

**Conclusion:**

Overall, DFUs were identified as critical predictors of mortality in people living with diabetes mellitus, mostly among those with type 2 diabetes mellitus. The impact of DFUs on mortality risk is multifaceted and context-dependent, highlighting the leading role of prior amputations. These findings underscore the need for targeted early interventions and the implementation of strategies aimed at reducing mortality in this high-risk population.

## INTRODUCTION

Diabetes mellitus (DM) is a chronic metabolic disorder characterized by persistent hyperglycemia resulting from altered insulin secretion, insulin resistance, or both (^1^). This condition is broadly classified into two main groups: type 1 (T1DM) and type 2 (T2DM).

The former is an autoimmune disease resulting from immune-mediated destruction of pancreatic beta cells, whereas the latter is primarily associated with insulin resistance and progressive beta-cell dysfunction (^1^).

The global burden of DM has increased steadily over the last decades (^1^). The 11th edition of the International Diabetes Federation Diabetes Atlas estimated that 589 million people are living with DM (PLWDM) around the world, with a projected 45% increase by 2050. This would mean that more than 853 million people will be affected, with Brazil being among the countries with the highest number of T1DM and T2DM cases (^1^).

The International Working Group on the Diabetic Foot defines a DM-related foot ulcer (DFU) as an ulcer occurring in an individual with current or previously diagnosed DM, typically accompanied by diabetic peripheral neuropathy (DPN) and/or peripheral artery disease (PAD) in the lower extremity (^2^). Ulcers arise from a combination of factors besides DPN and PAD, such as poor glycemic control, long disease duration, and mechanical stress, often exacerbated by inadequate footwear and reduced foot sensation (^3^).

Overall, DFUs significantly increase the risk of infections, lower limb amputations, and mortality, placing a considerable burden on affected individuals and health care systems (^2^,^3^). The lifetime incidence of DFUs among PLWDM ranges from 19% to 34% (^4^,^5^). Of those who develop DFUs, approximately 20% will require lower limb amputation, either minor (below the ankle), major (above the ankle), or both, and about 10% will die within one year of the initial DFU (^4^,^5^). A recent meta-analysis reported an overall five-year mortality rate following a DFU of approximately 50%, with cardiovascular disease and foot infections identified as leading causes of death (^6^). In addition, a North American study estimated that the direct annual costs of hospital treatment for DFUs in the United States range between USD 9 billion and USD 13 billion, excluding the costs incurred in managing DM itself (^7^).

Despite global evidence of the negative impact of DFUs, significant gaps remain in the literature regarding their burden and mortality impact in Brazil. Given the rising prevalence of DM and its complications in the country, understanding the local impact of DFUs is essential for designing effective public health strategies and clinical interventions. Therefore, this study aimed to evaluate the impact of DFUs on mortality risk in a Brazilian cohort of PLWDM and to determine whether the presence of DPN, PAD, and lower limb amputations further influences mortality risk.

## SUBJECTS AND METHODS

This study employed a retrospective, population-based epidemiological design using data retrieved from medical records of individuals followed at an association of PLWDM between 1990 and 2024. The sample consisted of patients from the city of Bauru (São Paulo State, Brazil) and its surrounding areas, ensuring representative population coverage. It comprised individuals from diverse socioeconomic levels who were treated at the association after referral by the local public health network or by private endocrinologists.

Data were classified as sociodemographic (age at death, sex, ethnicity [White, Black, Brown, Yellow, Indigenous], and socioeconomic status [very high, high, medium, low]), clinical (DM type, DFU, DPN, PAD, amputation), laboratory (fasting glycemia [FG], glycated hemoglobin (HbA1c), lipid profile (high-density lipoprotein [HDL], low-density lipoprotein [LDL], triglycerides [TG], total cholesterol [TC]), and therapeutic (adherence to diet, oral antidiabetic drugs [OAD], and insulin use).

Socioeconomic status and ethnicity were self-reported according to the criteria of the Brazilian Institute of Geography and Statistics (^8^). For statistical purposes, ethnicity was grouped as White and Non-white, and socioeconomic status as high and non-high. DM diagnoses were made in accordance with the criteria of the Brazilian Diabetes Society (^9^).

The DPN was defined by the presence of symptoms (numbness, tingling, and/or burning pain) and/or evidence of large fiber deficits: loss of protective sensation (assessed with the 10g Semmes-Weinstein monofilament SORRI®), reduced or absent vibration sensation (tested with a 128 Hz tuning fork), and/or reduced or absent reflexes (assessed with a neurological hammer). Peripheral artery disease was assessed by the presence of clinical signs (cold skin, pale skin, edema, skin thinning, or color changes), in combination with diminished or absent distal pulses (anterior and posterior tibial) and/or an ankle-brachial index less than 0.9, according to International Working Group on the Diabetic Foot criteria (^2^,^3^).

All laboratory tests were conducted at the same clinical analysis laboratory. Fasting glycemia values between 80 and 130 mg/dL, HbA1c below 7.0% for individuals under 60 years of age, and below 7.5% for those 60 years or older were considered on target according to American Diabetes Association and Brazilian Diabetes Society guidelines (^10^,^11^). Regarding lipids, LDL levels below 100, 70, and 50 mg/dL were considered on target for individuals with low, high, and very high cardiovascular disease risk, respectively. Additionally, HDL levels above 40 mg/dL in men and 50 mg/dL in women were considered on target. The TG levels below 150 mg/dL and TC below 200 mg/dL were considered on target according to the Brazilian Cardiology Society guidelines (^10^).

In this study, DFUs were treated as a binary variable (presence/absence). Ulcer severity, duration, infection status, and recurrence were not reported. The absence of severity stratification markedly limits the interpretation and comparison with other cohorts. Cox proportional hazards models were used to estimate the impact of selected variables on individual mortality risk. Variables were included individually or in multivariable analyses, and potential interactions were tested using interaction terms in the Cox regression model. The proportionality of hazards was assumed for all variables. Multivariate analysis was performed by associating DFUs, amputation, DPN, and PAD with patients' clinical and demographic characteristics. This approach was chosen to assess whether any of these covariates could adjust the impact of DFUs, amputation, DPN, and PAD on patient mortality.

The primary analytic focus was on evaluating the association between these variables and mortality, rather than including all variables simultaneously in a single model. Therefore, multicollinearity among these four variables was not formally assessed, which is acknowledged as a potential limitation given that correlations could exist and may affect interpretation of their individual effects.

Kaplan-Meier curves were generated to visualize survival distributions, and differences between groups were evaluated using the log-rank test. Hazard ratios (HRs) and 95% confidence intervals (CIs) were reported for all variables; HR > 1 indicated increased mortality risk; HR <1 indicated reduced risk; and HR = 1 indicated no effect. Two-sided p-values <0.05 were considered statistically significant. All statistical analyses were performed using R software (v. 4.4.2®). Survival analyses were conducted with the survival and survminer packages. Graphs, including KaplanMeier curves and risk tables, were created using gg-plot2 and related functions. This study was approved by the Research Ethics Committee of Bauru Dental School (protocol no. 37022120.4.0000.5417).

## RESULTS

The final study sample consisted of 382 individuals, with their characteristics detailed in **Table 1.** Patients with DFUs exhibited an 89% increased risk of mortality compared to those without ulcers (HR: 1.89; 95% CI: 1.31-2.73; *p* < 0.01; **Figure 1).** Individuals who had undergone amputation faced a 123% higher mortality risk than those without amputations (HR: 2.23; 95% CI: 1.22-4.08; *p* < 0.01; **Figure 2).** Additionally, DPN was associated with a 37% increase in mortality risk compared to those without DPN (HR: 1.37; 95% CI: 1.09-1.72; *p* < 0.01; **Figure 3).** Although patients with PAD had a 22% higher mortality risk compared to those without, this difference did not reach statistical significance (HR: 1.22; 95% CI: 0.76-1.97; *p* = 0.390). Individuals with DFUs not using OADs had a 165% higher risk of mortality than those without DFUs who were on these medications (HR: 2.65; 95% CI: 1.14-6.14; *p* = 0.023). Notably, patients with a history of amputation combined with inadequate FG levels exhibited a 41% higher risk of death (HR: 1.41; 95% CI: 1.25-1.88; *p* = 0.043). The other variables did not show a statistically significant impact on mortality **(Table 2).**

**Table 1:** Demographic, laboratory, and therapeutic characteristics of the sample

**Variable**	**Sample (n = 382)**
Demographic characteristics, n (%)	
Male	191 (49.99)
Female	190 (50.01)
White	317 (82.98)
Non-white	65 (17.02)
High socioeconomic class	67 (17.53)
Non-high socioeconomic class	315 (82.47)
Laboratory characteristics, n (%)	
FG on target	141 (36.91)
FG out of target	241 (63.09)
HbA1c on target	75 (19.63)
HbA1c out of target	307 (80.37)
HDL on target	153 (40.05)
HDL out of target	229 (59.95)
LDL on target	144 (37.69)
LDL out of target	238 (62.31)
TG on target	221 (57.85)
TG out of target	161 (42.15)
TC on target	218 (57.06)
TC out of target	164 (42.94)
Therapeutic approaches, n (%)	
Adherence to diet	351 (91.88)
Non-adherence to diet	31 (8.12)
Insulin use	91 (23.82)
Non-insulin use	291 (76.17)
AOD	218 (57.06)
Non-AOD use	164 (42.94)
AOD and insulin (simultaneous use)	54 (14.13)
Non-AOD and insulin (simultaneous use)	328 (85.87)

FG: fasting glycemia; HbA1c: glycated hemoglobin; HDL: high-density lipoprotein; LDL: low-density lipoprotein; TC: total cholesterol; TG: triglycerides; AOD: antidiabetic oral drugs.

**Table 2:** Statistical significance (*p*-value) of variables associated with mortality risk

**Variable**	**DFU**	**Amputation**	**DPN**	**PAD**
Sex	0.334	0.170	0.590	0.812
Ethnicity	0.428	0.214	0.278	0.712
Socioeconomic status	0.512	0.849	0.864	0.147
DM type	0.159	0.716	0.186	0.388
FG	0.975	0.007*	0.790	0.526
HbA1c	0.331	0.057	0.712	0.667
HDL	0.435	0.659	0.274	0.220
LDL	0.112	0.751	0.581	0.383
TG	0.673	0.277	0.634	0.227
TC	0.138	0.240	0.549	0.214
Diet	0.158	0.797	0.670	0.469
OAD	0.023	0.883	0.314	0.552
Insulin	0.900	0.934	0.164	0.563
AOD and insulin use	0.721	0.419	0.527	0.313

* Statistical significance. AOD: antidiabetic oral drug; DFU: diabetic foot ulcers; DM: diabetes mellitus type; DPN: diabetic peripheral neuropathy; PAD: peripheral artery disease; FG: fasting glycemia; HbA1c: glycated hemoglobin; HDL: high-density lipoprotein: LDL: low-density lipoprotein; TC: total cholesterol; TG: triglycerides.

**Figure 1:**
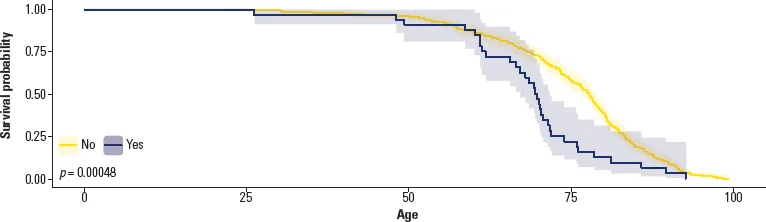
Survival probability according to the presence or absence of diabetic foot ulcers.

**Figure 2:**
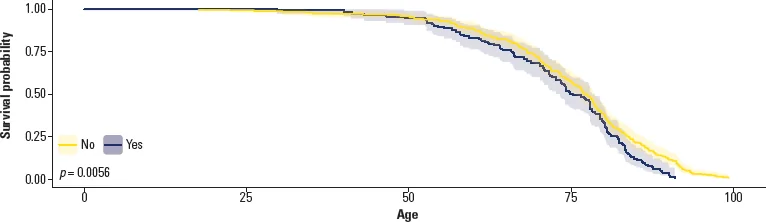
Survival probability according to the presence or absence of amputations.

**Figure 3:**
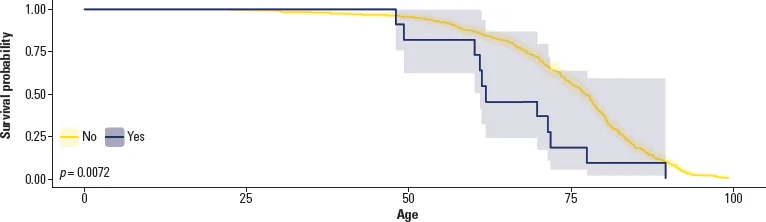
Survival probability according to the presence or absence of diabetic peripheral neuropathy.

## DISCUSSION

In this cohort of 382 participants, DFUs, amputations, and DPN were each associated with significantly elevated risks of death, with HRs of 1.89, 2.23, and 1.37, respectively. The PAD showed a modest but nonsignificant increase in mortality risk. The combined effect of complications and inadequate metabolic control was especially detrimental: patients with foot ulcers who did not receive OADs experienced a 165% higher risk of mortality, and those with prior amputations and poor FG had a 41% increased mortality risk. The remaining variables did not show statistically significant impacts on individual mortality risk.

Our study identified a significant association between DFUs and increased mortality risk. Consistently, a meta-analysis including over 446,000 individuals found that the presence of DFUs was associated with a 2.45-fold increase in all-cause mortality (^11^). This elevated risk is often attributed to prolonged inflammation, recurrent infections, and impaired wound healing characteristic of DFUs, which may exacerbate systemic inflammation and lead to multi-organ dysfunction (^11^). In contrast, a Danish nationwide cohort study reported no significant difference in mortality between individuals with and without DFUs, suggesting that ulceration alone may not determine increased mortality risk (^12^). Meanwhile, a French cohort study found that individuals with neuropathic DFUs had a five-year survival rate of 84% (^13^). These findings indicate that DFUs type and associated comorbidities may play critical roles in determining outcomes, beyond the mere occurrence of DFUs itself (^12^,^13^).

In this study, a history of amputation was associated with higher mortality risk. A United Kingdom cohort study reported a twofold increase in mortality risk among individuals after lower extremity amputations, especially within the first year post-amputation, potentially reflecting differences in the timing of amputation and baseline comorbidities (^14^). Similarly, a Brazilian study found that individuals with major amputations had an almost 1.8-fold higher mortality risk than those without amputations, noting that severity and comorbidities likely influence survival outcomes (^15^). In contrast, a German prospective cohort study found no significant increase in long-term mortality among individuals undergoing minor amputations when early multidisciplinary care was provided, emphasizing the importance of timely intervention (^16^).

The presence of DPN was associated with increased mortality risk in our study population. A retrospective study conducted in Nottingham (UK) observed that individuals with neuropathic osteoarthropathy had higher-than-expected mortality rates (^17^). Nonetheless, another study from the United Kingdom found that increased mortality in patients with DFUs occurred regardless of the presence of neuropathy (^18^).

Our results align with other studies that did not observe a significant association between PAD and increased mortality among PLWDM. For instance, the ADVANCE study found that, while macrovascular complications such as PAD were strongly associated with cardiovascular events, they did not independently predict all-cause mortality, as other factors (e.g., glycemic control, blood pressure, and renal function impairment) could account for this (^19^). Conversely, a decade-long cohort study in China observed that patients with severe PAD experienced more than a 3.5-fold increase in mortality (^20^).

We found that individuals with DFUs who were not using OADs experienced a significantly increased mortality risk. A North American study reported similar findings, with non-OADs users exhibiting approximately a 2.5-fold higher risk of death, supporting the reliability of this association (^21^). One possible explanation is that OADs may improve glucose control, reduce systemic inflammation, and prevent the progression of microvascular complications, thereby lowering mortality risk (^21^). Conversely, a Spanish study found no statistically significant difference in mortality risk between patients with DFUs who did and did not use OADs (^22^). Their study suggested that the severity of comorbid conditions, such as cardiovascular disease or renal insufficiency, as well as ulcer severity and access to wound care, may have diluted the effect of OADs use on survival (^22^).

An Australian study noted an increased mortality risk for patients with PAD and T2DM who were using insulin (^23^). In this relationship, insulin acts not as a direct cause, but rather the observed higher mortality is attributed to more advanced T2DM, since insulin therapy in these patients typically indicates longstanding poor glycemic control, a higher burden of comorbidities, and greater overall clinical complexity (^23^). Nevertheless, in our study, insulin use was not a statistically significant modifier of mortality risk. Similar findings were reported in a meta-analysis, which demonstrated that the presence of a DFU significantly increased mortality regardless of insulin use or DM type (^24^). These findings suggest that while insulin therapy may serve as a surrogate marker of disease severity in some contexts, its role as an independent predictor of mortality in patients with DFUs remains uncertain and may vary depending on population characteristics and study design (^24^).

Our study also observed that patients with a history of amputation and inadequate FG had a higher risk of death. Supporting this finding, a South Korean study reported that individuals with higher FG levels at hospital admission had an increased risk of both amputation and mortality (^25^). However, the relationship between FG and mortality appears complex and not strictly linear. Another South Korean study demonstrated that this association may follow a J-shaped curve, suggesting that both low and high FG levels are associated with increased mortality (^26^). Multiple clinical factors, including age, hypertension, sarcope-nia, coronary artery disease, and cerebrovascular disease, are likely to influence this relationship, highlighting that FG should not be interpreted in isolation, but rather in the broader clinical context (^26^). Although FG was used as a prognostic marker in our analysis, its role as a predictor of long-term mortality remains limited compared to more integrative measures of glycemic exposure.

A US study showed that PLWDM and DFUs with elevated HbA1c levels are at higher risk of developing new ulcers, undergoing lower limb amputations, and experiencing higher mortality (^27^). This association is biologically plausible, as chronic hyperglycemia contributes to impaired wound healing, increased susceptibility to infections, and the progression of microvascular and macrovascular complications (^27^). Nonetheless, HbA1c was not a significant predictor of mortality in our study. It is important to highlight evidence from a large cohort study involving British and Taiwanese populations, which demonstrated that individuals with a higher burden of comorbidities had lower HbA1c levels but a higher risk of mortality (^28^) suggesting that HbA1c alone may not be sufficient to capture mortality risk, particularly in more clinically complex patients (^28^).

Some studies have identified demographic, clinical, and laboratory parameters as important mortality predictors among individuals with DFUs. An Italian study observed that HDL levels were independent predictors of mortality in a cohort of patients with T2DM (^29^). Similarly, an Australian study noted that, after multivariate adjustment, age, chronic kidney disease, and low plasma albumin were independent predictors of mortality in individuals with DFUs (^30^). As observed in our study, one study found that DFU-related mortality was independent of demographic, clinical, and laboratory parameters (e.g., age, sex, HbA1c, and other variables) (^31^). These findings may be attributed to methodological variations such as population heterogeneity, endpoint definitions, and follow-up duration (^31^). Additionally, the influence of healthcare access and the timing of interventions may outweigh traditional risk factors in certain cohorts, thereby obscuring the expected relationships (^31^). As such, these studies have highlighted the necessity of context-aware analyses when interpreting the prognostic significance of clinical and laboratory data (^31^).

The economic and societal burden of DFUs is profound, with costs varying substantially across regions due to differences in healthcare infrastructure, service delivery models, and disease prevalence (^32^,^33^,^34^). Numerous studies have consistently demonstrated that individuals with DFUs incur markedly higher healthcare expenditures, primarily driven by extended hospital stays, complex surgical procedures, intensive wound management, and prolonged rehabilitation (^33^). Indeed, research from Iran, the US, and Turkey have reported that over USD 10 billion are spent annually on direct and indirect costs related to hospitalization for patients with DFUs, with these expenditures increasing exponentially in recent years (^32^,^33^,^34^). However, the magnitude of this financial burden is highly context-dependent (^32^), as high-income countries may partially mitigate costs through integrated care pathways, multidisciplinary foot care teams, and robust preventive programs that reduce hospitalizations and amputations (^32^). In low- and middle-income countries, the absence of subsidized care, coupled with limited access to specialized surgical interventions and preventive services, amplifies both the economic and human burden of DFUs (^33^,^34^).

Beyond economic considerations, DFUs have a profound impact on individuals' quality of life, affecting physical, psychological, and social well-being (^35^). Physically, individuals with DFUs often experience chronic pain, limited mobility, and an increased risk of infections and amputations, all of which significantly impair daily functioning and autonomy (^36^). Psychologically, the persistent nature of these ulcers, combined with the looming threat of limb loss, contributes to heightened levels of anxiety, depression, and emotional distress (^35^,^36^). Individuals with DFUs have reported markedly lower scores across multiple dimensions of health-related quality of life compared to both the general population and PLWDM without foot complications (^35^). Socially, the debilitating nature of DFUs can reduce participation in occupational and recreational activities, social isolation, and increase reliance on caregivers, thereby intensifying both personal and familial burdens (^36^). Furthermore, the stigma associated with visible wounds or amputations may further worsen self-esteem and social connectedness (^36^).

This study has several limitations that should be addressed. As a retrospective study based on medical records, it is inherently susceptible to information bias and missing data, which may impact the completeness and accuracy of the dataset. Additionally, the cross-sectional design did not allow inclusion of individuals with DFUs who died prematurely. Since PLWDM were recruited from a single center, our findings may not be fully generalizable to the broader Brazilian population, particularly those without access to specialized DM care. Data regarding the causes of death, as well as DFUs characteristics (e.g., severity, recurrence, and infection) were unavailable, limiting in-depth analysis on this subject. Hence, we suggest that future studies address this topic to contribute to a better understanding of the subject. In future research, it would be beneficial to perform clinical tests to diagnose small fibers and arteries alterations, rather than relying solely on clinical data, to assess whether this alters the total prevalence of neuropathy and vasculopathy and their relationship with mortality.

In conclusion, this study demonstrated that the presence of DFUs is a critical determinant of increased mortality risk in PLWDM, particularly among those with T2DM. The impact of DFUs on mortality risk is complex and dependent on several factors, including demographic, social, and economic considerations. The results highlight the urgency of implementing targeted interventions and early detection strategies for DFUs to improve prognosis, reduce mortality, and enhance quality of life for PLWDM.

## Data Availability

any data pertaining to this study will be available upon request to the corresponding author.
